# Two Tandem RNase III Cleavage Sites Determine *betT* mRNA Stability in Response to Osmotic Stress in *Escherichia coli*

**DOI:** 10.1371/journal.pone.0100520

**Published:** 2014-06-23

**Authors:** Minji Sim, Boram Lim, Se-Hoon Sim, Daeyoung Kim, Euihan Jung, Younghoon Lee, Kangseok Lee

**Affiliations:** 1 Department of Life Science, Chung-Ang University, Seoul, Republic of Korea; 2 Department of Chemistry, KAIST, Daejeon, Republic of Korea; Centre National de la Recherche Scientifique, Aix-Marseille Université, France

## Abstract

While identifying genes regulated by ribonuclease III (RNase III) in *Escherichia coli*, we observed that steady-state levels of *betT* mRNA, which encodes a transporter mediating the influx of choline, are dependent on cellular concentrations of RNase III. In the present study, we also observed that steady-state levels of *betT* mRNA are dependent on RNase III activity upon exposure to osmotic stress, indicating the presence of *cis*-acting elements controlled by RNase III in *betT* mRNA. Primer extension analyses of *betT* mRNA revealed two tandem RNase III cleavage sites in its stem-loop region, which were biochemically confirmed via *in vitro* cleavage assays. Analyses of cleavage sites suggested the stochastic selection of cleavage sites by RNase III, and mutational analyses indicated that RNase III cleavage at either site individually is insufficient for efficient *betT* mRNA degradation. In addition, both the half-life and abundance of *betT* mRNA were significantly increased in association with decreased RNase III activity under hyper-osmotic stress conditions. Our findings demonstrate that *betT* mRNA stability is controlled by RNase III at the post-transcriptional level under conditions of osmotic stress.

## Introduction

RNase III plays an important role in regulating gene expression by degrading and processing mRNA in both prokaryotes and eukaryotes [1], [2]. The RNase III family enzymes specifically cleave double-stranded RNA (dsRNA), creating 5′-phosphate and 3′-hydroxyl termini with a two-nucleotide overhang. In *Escherichia coli*, RNase III, encoded by the *rnc* gene, consists of a ribonuclease domain (amino acid residues 21–149) and a dsRNA-binding domain (residues 155–209). *E. coli* RNase III functions as a homodimer in which two ribonuclease domains form a single processing center, and each domain contributes to the cleavage of one RNA strand of the duplex substrate [3], [4].

Several *E. coli* mRNA transcripts have been identified whose abundance is dependent on the cellular concentration and activity of RNase III, including *rnc* [5], *pnp* [6], *bdm* [7], *corA* [8], *proU* [9], *mltD* [10], and *rng* [11] mRNA. A recent genome-wide analysis of such RNase III concentration-dependent transcripts indicated that the stability of a large number of mRNA transcripts can be controlled by RNase III [7]. This study further identified a subgroup of mRNA transcripts encoding factors associated with the osmosensing of *E. coli* that are downregulated by RNase III. Among them, the mechanisms of RNase III-mediated regulation of *bdm* [7] and *proU* [9] genes have been identified, which revealed that RNase III activity on *bdm* and *proU* mRNA is significantly altered in *E. coli* subjected to hyper- and hypo-osmotic stress. These results indicate that RNase III activity is regulated by external signals in *E. coli*. However, *betT*, another important osmoregulator, has not been studied with respect to RNase III-mediated regulation upon osmotic stress although *betT* mRNA abundance was found to be dependent on cellular RNase III concentrations.

The *betT* gene forms part of the osmoregulatory system Bet regulon, which participates in the synthesis of glycine betaine from externally supplied choline. The Bet system is composed of the following: BetA, a choline dehydrogenase; BetB, a betaine aldehyde dehydrogenase; BetI, a regulatory protein; and BetT, a choline transporter [12]–[15]. BetT protein belongs to the betaine-choline-carinitine transporter family and has been characterized as a ubiquitous proton-coupled transporter for choline. The functional role of BetT in the regulation of osmolarity is well studied. BetT mainly transports external choline into cells with high affinity (*K*m = 8 µM) when the external concentration of choline is low [16]. At high choline concentrations (*K*m = 1.5 mM), choline uptake is mediated by the ProU transport system [17]. However, the functional correlation between the Bet regulon and the ProU transport system in the regulation of cellular osmolarity is unclear [16]–[18].

The physiological role of BetT in osmotic stress resistance has been previously reported [16]; however, the regulation mechanisms of *betT* gene expression and the factors involved have not been fully characterized. In the present study, we investigated the effects of RNase III activity on *betT* gene expression in *E. coli*. Our results provide direct evidence that RNase III controls the degradation of *betT* mRNA by cleaving at two sites in its open reading frame region, thereby suggesting a physiological relationship between the regulation of RNase III activity and osmotic stress resistance.

## Materials and Methods

### Strains and plasmids

Construction of the *E. coli* strain MG1655*rnc* has been previously described [8]. To overexpress *betT*, a DNA fragment encoding *betT* was amplified using the primers betT-5′ (5′-ATGCGGCCGCGAATTTGATTTTAAATAGTA, in which the transcriptional initiation site of the 5′-*betT* gene is underlined) and betT-3′ (5′-ATGCGGCCGCTCACGCGTCCGGGAACATCA, in which the complementary sequence of the translational stop codon of the *betT* open reading frame is underlined), and cloned into the *Not*I site in pCAT924 [7], [19], [20]. The resulting plasmid (pBetRS1) overexpresses *betT* under the control of a constitutive *trp^c^* promoter. To express *betT* mRNA containing a single nucleotide substitution at the RNase III cleavage sites (C33U and C39U), pBetRS1-C33U and pBetRS1-C39U were constructed as follows: DNA fragments were amplified using overlap extension polymerase chain reaction (PCR) method and cloned into the *Not*I site in pCAT924. The primers used were betT-5′, betT-48F (5′-GGTGTTTTACACCTCCGCCG), betT-1610R (5′-TAATTCATCAGGCGCGAGAG), and betT-11D-67R (5′-CGGCGGAGG TGTAAAACACCACCGGATTGATTTTATCCTTTTCCCTGCTGTGTGAAAG, in which the mutated nucleotide is underlined) for pBetRS1-C33U, and betT-5′, betT-48F, betT-1610R, and betT-13I-67R (5′-CGGCGGAGGTGTAAAACACCACCGGATTAATTTTGTCCTTTTCCCTGCTG, in which the mutated nucleotide is underlined) for pBetRS1-C39U. The MG1655*betT*, MG1655*proV*, and MG1655*betTproV* strains were constructed via one-step inactivation of the chromosomal gene by using the method described by Datsenko and Wanner [21]. The primers used to construct *betT*-deleted strains were betT-H1 (5′-CATATGCAGACATGGCGCGGTTTTATGCAATAACAAGTGTAGGCTGGAGCTGCTTC, in which the complementary a 36 nt-long sequence of the *betT* 3′-untranslated region (UTR) is underlined) and betT-H2 (5′-GATTTTAAATAGTAACAATAACAGTGGGGATACTGGCATATGAATATCCTCCTTA, in which a 36 nt-long sequence of the *betT* 5′-UTR is underlined) with pKD3 as a template. The primers proV-5′-UTR-Km (5′-TATCGACATAGACAAATAAAGGAATCTTTCTATTGCAGAGCGCTTTTGAAGCTCAC, in which the sequence of the *proV* 5′-UTR is underlined) and proV-3′-UTR-Km (5′-TGGCGTGGTATCCCACGGATTATTTTGATCAGCCATCCCTTATTAGAAGAACTCGT, in which the complementary sequence of the *proV* 3′-UTR and the coding region is underlined) were used to amplify the kanamycin antibiotic resistance marker in pKD13 for construction of *proV*-deleted strains.

### Reverse Transcription (RT)-PCR analysis

RT-PCR analysis was performed as previously described [22], [23]. Total RNA was prepared using an RNeasy Miniprep kit (Qiagen). The primers used for *betT* were betT-F (5′-ATGACAGACCTTTCACACAG) and betT-652R (5′-TAAGCTGCACCACACCGATA). The primers used for M1 were rnpB-F (5′-GAAGCTGACCAGACAGTCGC) and rnpB-R (5′-AGGTGAAACTGACCGATAAG).

### *In vitro* cleavage analysis

His-tagged RNase III purification and cleavage assays were performed as previously described [1]. Template DNA fragments for the synthesis of full-length *betT* mRNA and the model hairpin RNA, which spans nucleotides 21–95 in the *betT* coding region (the first nucleotide of the start codon is at position 1) and encompasses the RNase III cleavage sites, were obtained by amplifying the corresponding sequence in pBetRS1 using the following primers: T7-betT-F (5′-TAATACGACTCACTATAGGAATTTGATTTTAAATAGTA , in which the 5′-UTR of *betT* is underlined) and betT-R (5′-TCACGCGTCCGGGAACATCA, in which the complementary sequence of the stop codon is underlined) for a full-length *betT* transcript, as well as T7-betT-21F (5′-TAATACGACTCACTATAGCAGGGAAAAGGACAAAATCAA , in which the 5′ region of model hairpin RNA is underlined) and betT-95R (5′-GTTGTCAGGGAAAACAACAAA , in which the complementary sequence of the 5′ region of model hairpin RNA is underlined) for the model hairpin RNA. Labeled RNA (0.2 pmol) was incubated with 1 ng of purified RNase III in the presence of 0.25 mg ml^−1^ of yeast tRNA (Ambion) and 20 U of RNase inhibitor (Takara) in 20 µl of cleavage buffer (30 mM Tris-HCl, pH 7.9, 160 mM NaCl, 0.1 mM dithiothreitol, 0.1 mM ethylenediamine tetraacetic acid (EDTA), pH 8.0). Cleavage reactions were initiated by adding 10 mM MgCl_2_ at 37°C. Samples were removed at various time intervals (0, 1, 2, and 4 min) and mixed with an equal volume of gel-loading buffer (deionized-formamide 95%, EDTA (pH 8.0), 0.025% sodium dodecyl sulfate, 0.025% xylene cyanol, and 0.025% bromophenol blue). The samples were denatured at 65°C for 10 min and separated on an 8% or 10% polyacrylamide gel containing 8 M urea and 0.5×Tris-borate EDTA.

### Primer extension

Primer extension analysis was performed using total RNA purified via heated phenol extraction and ethanol precipitation. The following 5′-^32^P-labeled primers were used: betT-120R (5′-GGCCGAGAAGTCGCGAAACA) and betT-273R (5′-TGAATTCCGGTTTGGATTGT). RNA with labeled primers were annealed at 65°C for 15 min, slowly cooled down to room temperature for 2.5 h, and extended at 42°C for 1 h using AMV reverse transcriptase (New England Biolabs). The extended fragments were denatured at 95°C for 10 min and separated on 10% polyacrylamide gels containing 8 M urea.

### Northern blot analysis

Forty micrograms of the total RNA sample prepared as describe above was denatured at 65°C for 10 min in twice the volume of formamide loading buffer and separated by electrophoresis on a 1.2% GTG agarose gel containing 2.3% formaldehyde. The gels were transferred onto nylon membranes (Hybond-XL, GE Healthcare) in 20× standard saline citrate. The random hexamer probes used for *betT* mRNA detection were synthesized using a random-primed DNA labeling kit (Roche; Pleasanton, CA), in which the PCR products containing the coding region of *betT* were used as a template. The primers used were betT-F and betT-R. The oligonucleotide probe used for M1 was rnpB-137R (5′-GCTCTCTGTTGCACTGGTCG).

### β-Galactosidase assays

β-Galactosidase activity in whole cells was determined as previously described [24].

## Results

### RNase III negatively regulates the expression of *betT*

**Figure 1:**
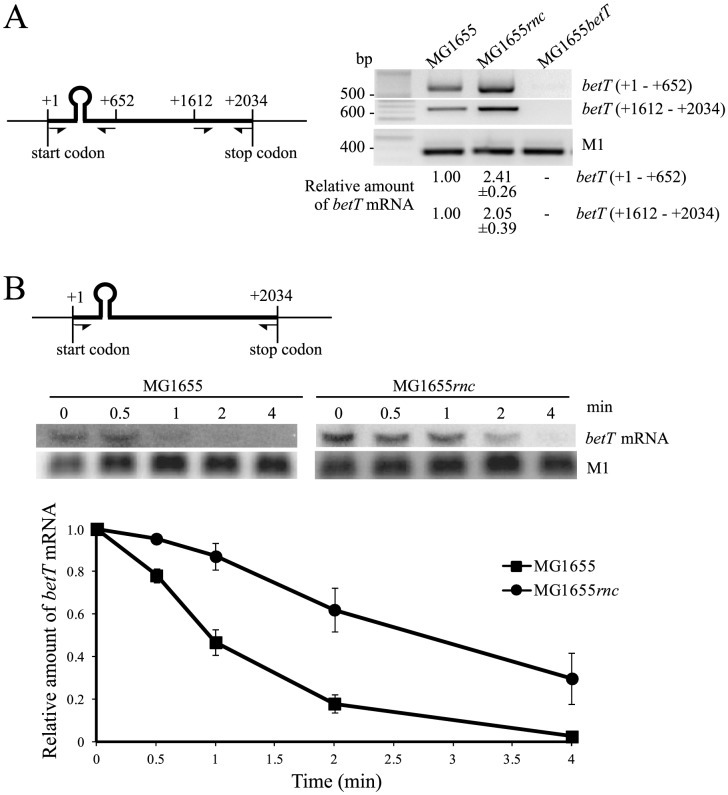
Downregulation of *betT* expression by RNase III. (A) Effects of RNase III on steady-state levels of *betT* mRNA. Total RNA samples were prepared from the cultures of *E. coli* strains MG1655, MG1655*rnc*, and MG1655*betT* grown at 37°C in LB medium until an OD_600_ of 0.6 was reached. Steady-state levels of *betT* mRNA were determined by semi-quantitative RT-PCR analysis. (B) Effects of cellular RNase III concentration on *betT* mRNA decay demonstrated by northern blot analysis. *E. coli* strains MG1655 and MG1655*rnc* were grown as described above and total RNA samples were prepared from at 0 min, 0.5 min, 1 min, 2 min, and 4 min after the addition of rifampicin (1 mg ml^−1^). The relative amounts of *betT* mRNA transcripts were measured by setting the amount of *betT* mRNA in MG1655 cells (A) or in cultures 0 min after the addition of rifampicin (B) to 1, and plotted in the graphs. Amounts of full-length M1 RNA, which served to normalize the amount of *betT* mRNA in each RNA sample, were determined by semi-quantitative RT-PCR (A) or northern blot (B). The primers used for RT-PCR and PCR DNA for the production of random probe are shown with numbers indicating nucleotide positions in *betT* mRNA (the first nucleotide of the start codon as +1). The experiments were repeated three times and averaged. The error bars (standard errors of the mean) were used to indicate the range of assay results. The half-lives of *betT* mRNA were estimated by fitting and extrapolating the plots in the graphs.

To investigate whether the absence of RNase III affects the abundance of *betT* mRNA, we measured the steady-state levels of *betT* mRNA in wild-type and *rnc*-mutant *E. coli* by using semi-quantitative RT-PCR. Two sets of primers were used to amplify cDNAs (+1 to +652 and +1612 to +2034) (Figure 1A). Consistent with the microarray data from our previous study [7], *rnc*-mutant cells showed a 2.1–2.4-fold increase in the amount of *betT* mRNA compared to that observed in wild-type cells (Figure 1A). To further explore the effects of RNase III concentration on *betT* mRNA decay, the half-life of *betT* mRNA was evaluated by a northern blot analysis (Figure 1B). The half-life of the *betT* mRNA expressed in *rnc*-mutant cells was determined to be two-fold higher than that expressed in wild-type cells. Analogous results were obtained when the half-life of *betT* mRNA was measured by a semi-quantitative RT-PCR analysis (Figure S1). For these experiments, M1 RNA, the 377 nt catalytic component of the t-RNA processing ribozyme RNase P, was used as an internal control because it is a stable RNA whose half-lives are 50–60 min in the exponential phase of growth [25]. These results indicate that both the steady-state level and half-life of *betT* mRNA correlate with the cellular RNase III concentration, indicating the involvement of RNase III in *betT* mRNA decay.

### Identification of RNase III cleavage sites in *betT* mRNA

**Figure 2:**
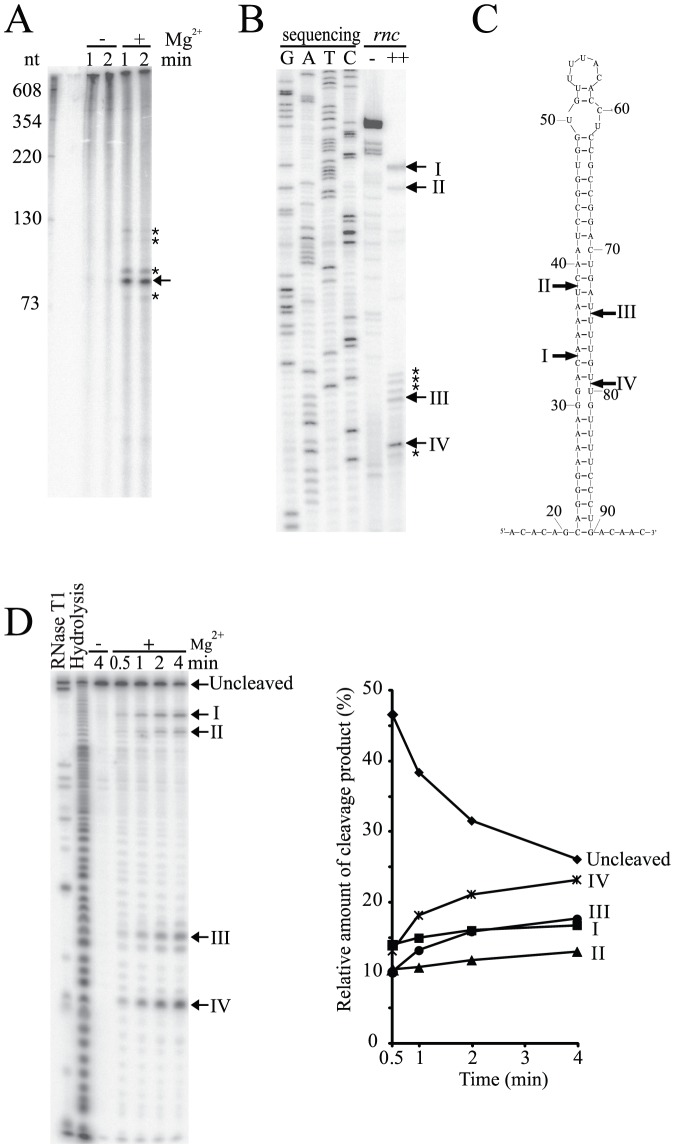
Identification of RNase III cleavage sites in *betT* mRNA *in vitro* and *in vivo*. (A) *In vitro* cleavage of the full-length synthetic *betT* mRNA. The 5′-end-labeled *betT* transcript (4 pmol) was incubated with purified RNase III (1 pmol) in cleavage buffer with (+) or without (-) MgCl_2_ at 37°C. The size of the cleavage products was estimated using size markers generated by internally labeled transcripts. The major cleavage products are indicated with arrows. Other minor cleavage products are indicated with asterisks. (B) Primer extension analysis of *betT* mRNA. Total RNA was prepared from MG1655*rnc* harboring pBetRS1 and either pKAN6B or pRNC1, which exogenously overexpressed *betT* mRNA, hybridized with a 5′-^32^P-end-labeled primer (betT+120R), and extended using AMV reverse transcriptase. Sequencing ladders were produced using the same primer used in cDNA synthesis and PCR DNA, encompassing the *betT* gene as a template. (C) The predicted secondary structure of *betT* mRNA region encompassing RNase III cleavage sites. The secondary structure was determined using the M-fold program [33]. (D) *In vitro* cleavage of the model hairpin RNA of *betT* mRNA. 3′-end-labeled *betT* model hairpin (25 pmol) was incubated with purified RNase III (1 pmol) in a cleavage buffer with (+) or without (−) MgCl_2_, respectively. Cleavage products (I, II, III, and IV) were identified using size markers generated by alkaline hydrolysis and RNase T1 digestion. Relative abundance of each cleavage product was assessed by measuring the radioactivity of each band and plotted.

To investigate whether *betT* mRNA contains *cis*-acting elements that are responsive to RNase III, we performed an *in vitro* cleavage assay using purified RNase III and synthetic full-length *betT* mRNA transcripts. RNase III cleavage of 5′-end labeled *betT* mRNA generated a major cleavage product that was approximately 80 nt in length and several minor cleavage products whose lengths were approximately 70–130 nt (Figure 2A). This result indicates that RNase III recognizes and cleaves specific regions of *betT* mRNA.

In order to identify the RNase III cleavage sites in *betT* mRNA, we performed primer extension analysis using a 5′-end-labeled primer (betT+273R) that was designed to bind to a downstream region of the RNase III cleavage sites that were deduced from the *in vitro* cleavage assay. Because *betT* expression is induced at high salt concentrations, total RNA was purified from wild-type and *rnc*-mutant cells grown in plain (0.17 M NaCl) or 0.50 M NaCl-supplemented Luria-Bertani media. However, we were not able to observe cDNA bands extended from *betT* mRNA in all reaction mixtures (Figure S2, lanes 5–8). This result indicated that the expression levels of *betT* mRNA were not high enough to be detected using primer extension analysis. Thus, the reaction was performed with the total RNA prepared from *E. coli* cells overexpressing *betT* mRNA and RNase III, and we observed several cDNA bands extending from *betT* mRNA that appeared to be RNase III-dependent (Figure S2, lane 10). These bands were not present in the lane loaded with the reaction mixture containing total RNA extracted from cells that overexpressed *betT* mRNA in the absence of RNase III expression. To obtain a higher resolution of the cDNA bands, we used another 5′-end-labeled primer (betT+120R) for primer extension analysis. We observed four distinct cDNA bands that were dependent on RNase III (Figure 2B, lane 5 vs. lane 6). These bands corresponded to sites that are located in the double-stranded region of the *betT* mRNA coding sequence, and the cleavage of these sites by RNase III was predicted to produce cleavage products with a two-nucleotide overhang at the 3′-end, which is a property of RNase III cleavage. These putative tandem RNase III cleavage sites were designated as sites I–IV (Figure 2C). Four additional minor cDNA bands were also observed within close proximity of sites III and IV (Figure 2B).

To biochemically demonstrate the cleavage of *betT* mRNA by RNase III, an *in vitro* cleavage assay was performed using a model hairpin RNA of *betT* mRNA containing RNase III cleavage sites I–IV in *betT* mRNA (Figure 2C). *In vitro* RNase III cleavage of a 3′-end-labeled model hairpin RNA generated four major cleavage products. The lengths of the major products corresponded to cleavage sites identified from primer extension analyses (Figure 2D). We also detected other minor cleavage products that were likely produced by the random cleavage of transcripts by RNase III, which is an intrinsic property of RNase III *in vitro* [7], [8], [26]. We observed that the cleavage products at sites III and IV accumulated at similar rates, indicating that the RNase III cleavage at two tandem sites (I and IV; II and III) was independent (Figure 2D).

### RNase III cleavage determines *betT* mRNA stability *in vivo*

**Figure 3:**
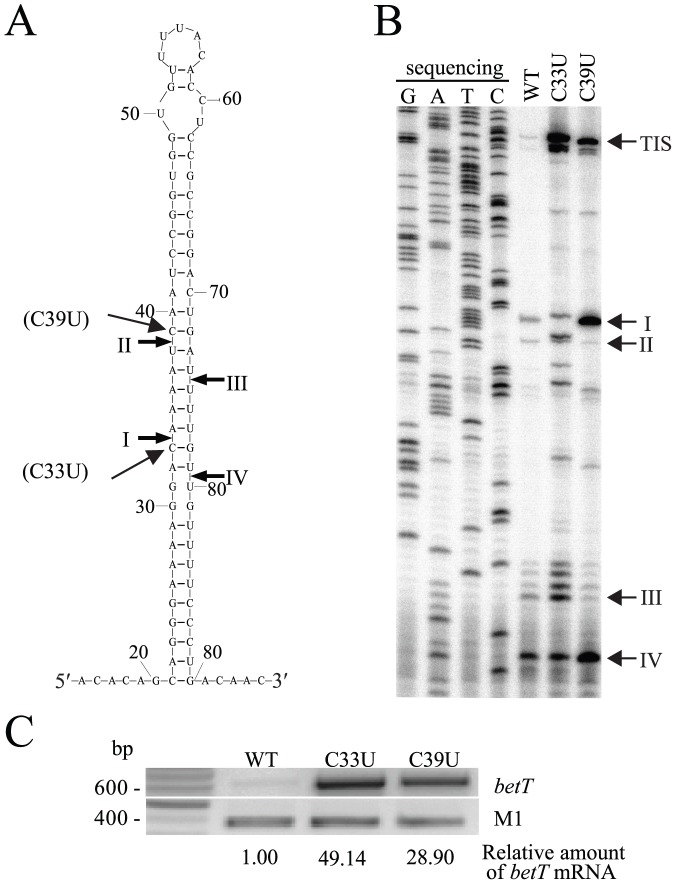
Inhibition of RNase III cleavage of *betT* mRNA by introducing a mutation at the cleavage site. (A) Secondary structures of the hairpin encompassing RNase III cleavage sites. Nucleotide substitutions (C33U and C39U) at the RNase III cleavage sites are indicated. (B) Effects of a nucleotide substitution at the cleavage sites on RNase III activity on *betT* mRNA. Primer extension experiments were performed as described in the legend to Figure 2B. (C) Effects of an RNase III cleavage site mutation on steady-state levels of *betT* mRNA determined by semi-quantitative RT-PCR analysis. Total RNA was prepared from strains MG1655*rncbetT* harboring pRNC1 and either pBetRS1 or its derivative (pBetRS1-C33U or pBetRS1-C39U), which were grown in LB at 37°C to an OD_600_ of 0.6.

To test whether RNase III activity on cleavage sites regulated the stability of *betT* mRNA, we introduced a nucleotide substitution on cleavage site I or II (C33U or C39U) in the *betT* overexpression plasmid (pBetRS1) (Figure 3A). These mutations did not alter the secondary structure of *betT* mRNA or the subsequent amino acid sequence. Total RNA was isolated from the MG1655*rncbetT*-harboring pRNC1 plasmid and either pBetRS1 or a derivative of pBetRS1 (pBetRS1-MT), and primer extension analysis was performed. Whereas the cDNA bands corresponding to RNase III cleavage sites I–IV were clearly visible in the reaction mixture containing wild-type *betT* mRNA, the abundance and patterns of cDNA products from mutant *betT* mRNAs differed from those of wild-type *betT* mRNA (Figure 3B). Nucleotide substitution at site II (C39U) dramatically reduced RNase III cleavage activity at sites II and III, whereas the C33U mutation resulted in inhibition of RNase III cleavage at the mutated site and alterations of cleavage patterns. Despite the differences in the effects of these cleavage site mutations on RNase III activity on *betT* mRNA, the ratios of the total intensity of the cDNA bands corresponding to the RNase III cleavages sites to those corresponding to the transcriptional initiation site (TIS) were greatly decreased when these mutations were introduced (5.0-fold and 4.7-fold decrease for the C33U and C39U mutation compared with that of the wild-type, respectively), resulting in the significant accumulation of uncleaved mutant *betT* mRNAs. RT-PCR analysis of *betT* mRNA further confirmed increased steady-state levels of *betT* mRNA resulting from the mutations (Figure 3C). These results suggest that the inactivation of either dimeric cleavage site (I and IV or II and III), which is handled by a single processing center formed by dimeric RNase III [4], led to an increase in the *in vivo* stability of *betT* mRNA.

### Osmoregulation of *betT* expression by RNase III

**Figure 4:**
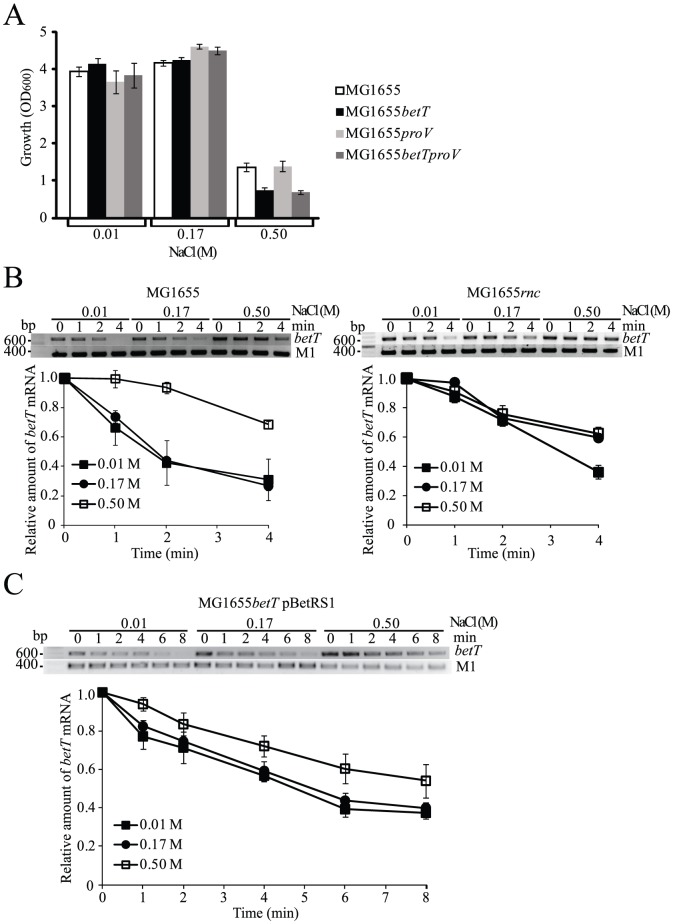
Osmoregulation of *betT* mRNA degradation by RNase III. (A) Effects of *betT* deletion on *E. coli* growth upon osmotic stress. The cultures of wild-type and *betT* and/or *proV*-deleted MG1655 were grown in M63 supplemented with 22 mM glucose until an OD_600_ of 0.3 was reached, and either treated with 0.01 M (hypo-osmotic), 0.17 M (normal), or 0.50 M (hyper-osmotic) NaCl with 10 µM choline. Growth was measured by analyzing the cell density (OD_600_) of cultures grown for 18 h. (B) Effects of osmotic stress on the half-life of *betT* mRNA. Total RNA was isolated from the cultures of MG1655 and MG1655*rnc* grown under the same conditions as described above, except that they were grown until an OD_600_ of 0.6 was reached followed by the addition of rifampicin (1 mg ml^−1^). (C) Effects of the *betT* promoter on the half-life of *betT* mRNA. MG1655*betT* harboring pBetRS1 was grown as described above. Semi-quantitative RT-PCR analysis was performed to measure the relative amounts of *betT* mRNA. Experiments were performed in triplicate and repeated at least three times.

Previous studies have demonstrated that RNase III activity is regulated by osmolarity within *E. coli* cells [7] and that BetT plays functional roles in the regulation of cellular osmolarity under low choline conditions [18], [27], [28]. These observations led us to investigate whether reduced RNase III activity caused by high osmolarity increases the stability of *betT* mRNA. First, we confirmed the functional role of *betT* in *E. coli* growth by culturing cells under hypo-osmotic (0.01 M NaCl), normal (0.17 M NaCl), and hyper-osmotic (0.50 M NaCl) conditions in the presence of low choline concentration (10 µM) (Figure 4A and S3). The *betT*-deleted *E. coli* cells showed reduced growth rates compared to those of wild-type cells under hyper-osmotic conditions, indicating the importance of *betT* expression under hyper-osmotic stress with low choline concentration as previously shown [16]. The growth of *proV*-deleted bacterial cells was comparable to that of wild-type cells as previously reported [27]–[30]; the ProU transport system is involved in the osmotic stress response under high choline conditions.

Next, we measured the half-lives of *betT* mRNA under different osmotic stress conditions. The results from semi-quantitative RT-PCR analysis showed that the decay rate of *betT* mRNA in wild-type *E. coli* under the hyper-osmotic condition was approximately two times lower than those under normal or hypo-osmotic conditions (Figure 4B, left panel). Steady-state levels of *betT* mRNA were also approximately two times higher under the hyper-osmotic condition, showing a correlation between the half-life and abundance of *betT* mRNA. The decay rates of *betT* mRNA in *rnc*-mutant cells under normal and hyper-osmotic conditions were similar with that in in wild-type *E. coli* under the hyper-osmotic condition (Figure 4B, right panel), indicating that decreased RNase III activity is associated with a prolonged half-life of *betT* mRNA in wild-type *E. coli* under the hyper-osmotic condition. Steady-state levels of *betT* mRNA in *rnc*-mutant cells were not significantly changed, indicating that transcriptional activation of *betT* mRNA expression did not occur under the hyper-osmotic condition that used in this study (Figure 4B). We also observed that *betT* mRNA decays faster, especially between 2 min and 4 min after inhibiting transcription by the addition of rifampicin to the cultures, in *rnc*-mutant cells under the hypo-osmotic condition (Figure 4B, right panel). Although further study is needed to explain this phenomenon, we think that *betT* mRNA decays via an unknown pathway in the absence of RNase III activity under hypo-osmotic condition. Nonetheless, these results indicate that increased steady-state levels of *betT* mRNA are mainly associated with decreased RNase III activity under conditions of hyper-osmotic stress. We further measured the half-lives of *betT* mRNA in strains that express *betT* mRNA from an osmotic stress-insensitive, constitutive promoter. MG1655*betT* harboring pBetRS1 was used for these experiments. The half-life of *betT* mRNA in MG1655*betT* harboring pBetRS1 under the hyper-osmotic condition was approximately 1.5 times higher than those under normal or hypo-osmotic conditions (5.1 min vs. 7.6 min) (Figure 4C). Steady-state levels of *betT* mRNA were also approximately 1.7 times higher under the hyper-osmotic condition, once again showing a correlation between the half-life and abundance of *betT* mRNA. These results indicate that the *betT* mRNA decay is controlled by RNase III in response to osmotic stress. We confirmed that RNase III activity is downregulated under hyper-osmotic conditions using the *E. coli* strains KSC004 and KSC006. These *E. coli* strains contain an RNase III target site in single copy of a *pnp′-′lacZ* (KSC004) or an *rnc′-′lacZ* (KSC006) reporter gene [31], [32]. Following a hyper-osmotic shift with 0.50 M NaCl, the RNase III activity in the KSC004 strain decreased by approximately 2.8-fold relative to cells that were under hypo-osmotic (0.01 M NaCl) or normal (0.17 M NaCl) conditions (Figure S4). Analogous results were obtained with the KSC006 strain. The expression levels of RNase III protein did not significantly change under different osmotic conditions (Figure S4), which is in agreement with the study conducted by Sim *et al*. [7]. Our results indicate that RNase III activity under hyper-osmotic stress conditions was downregulated, which is associated with an increase in the stability of *betT* mRNA.

## Discussion

Although the factors involved in osmoregulation mechanisms in *E. coli* have been well studied, the regulation mechanisms underlying rapid control of osmosensing factors are not fully understood. Recent studies have demonstrated that RNase III plays roles in the regulation of several stress response factors, such as the biofilm-dependent modulation protein (Bdm) [7], osmosensing transporter protein (ProU) [9], and cobalt resistance protein (CorA) [8], at the post-transcriptional levels in *E. coli*. The present study showed that under conditions of hyper-osmotic stress, *betT* expression is also regulated by RNase III at the post-transcriptional level.

Given that RNase III enzymes require a minimum of a 22-bp stem for cleavage activity, RNase III cleavage at one dimeric site can abolish RNase III cleavage activity at the other dimeric site in *betT* mRNA. The results of the *in vitro* cleavage assay support this notion as the cleavage products of the 3′-^32^P-end-labeled model hairpin RNA that were generated by RNase III cleavage at sites proximal to the 3′-end of the transcript (III and IV) appear to accumulate at similar rates, indicating that the RNase III cleavage at the two tandem sites (I and IV; II and III) is independent (Figure 2D). In addition, the primer extension analysis on *betT* mRNA also indicated a correlation in the intensity of the cDNA bands between the two cleavage sites (I and IV; II and III). These results demonstrate that the RNA fragments generated by RNase III cleavage at one dimeric RNase III site in *betT* mRNA are not likely to be further cleaved by RNase III, indicating the independent cleavage of two tandem RNase III sites in *betT* mRNA. This result raises the question as to why *betT* mRNA contains two consecutive RNase III cleavage sites, neither of which is sufficient on its own for efficient *betT* degradation. This appears to be a unique property, as no other known RNase III substrates show two tandem RNase III cleavage sites. One possibility is that both sites are cryptic and do not provide the structural determinants that are required for full RNase III activity. This possiblity is supported by our mutational analyses at the *betT* mRNA cleavage sites, which demonstrated that inactivation of RNase III cleavage at either site (I and IV or II and III) was sufficient to inhibit *betT* mRNA degradation (Figure 3).

Similar to two other RNase III-controlled mRNA species that encode factors responsive to osmotic stress, our findings show that steady-state levels of *betT* mRNA are dependent on RNase III activity under conditions of osmotic stress (Figure 4C). The correlation between the increased half-life of *betT* mRNA and decreased RNase III activity under hyper-osmotic conditions (Figure 4) further implicates the osmoregulation of RNase III activity on *betT* mRNA. It has been reported that RNase III activity can be regulated in *E. coli* under other conditions such as bacteriophage infection and cold- or aminoglycoside antibiotic-stress [11], [32], [34]. Protein regulators for RNase III have been also identified: Bacteriophage T7 protein kinase activates RNase III by phosphorylating the enzyme on serine [34] and YmdB inhibits dimerization of the enzyme [32]. However, these protein regulators do not appear to be involved osmoregulation of RNase III activity [7]. An unknown signal may be triggered by exposure of *E. coli* to hyper-osmotic stress that inactivates RNase III, thereby inhibiting the digestion of *betT* mRNA, resulting in increased cellular expression of BetT. Although the exact mechanism for the downregulation of RNase III activity under hyper-osmotic stress is unknown, we speculate that there is an RNase III-mediated osmoregulatory mechanism by which the expression levels of osmosensing factors, including Bdm, ProU, and BetT, are rapidly balanced out via the modulation of RNase III activity upon exposure to osmotic stress.

## Supporting Information

Figure S1 Effects of RNase III on the half-life of *betT* mRNA. Total RNA was isolated from the cultures of MG1655 and MG1655*rnc* grown at 37°C in LB medium until an OD_600_ of 0.6 was reached followed by the addition of rifampicin (1 mg ml^−1^). Semi-quantitative RT-PCR analysis of the cDNA +1-+652 was performed to measure the relative amounts of *betT* mRNA by setting the amount of *betT* mRNA in cultures 0 min after the addition of rifampicin to 1. The experiments were repeated three times and averaged. The error bars (standard errors of the mean) were used to indicate the range of assay results. The half-lives of *betT* mRNA were estimated by fitting and extrapolating the plots in the graphs.
(EPS)

Figure S2 Primer extension analysis of *betT* mRNA. Total RNA was prepared from MG1655, MG1655*rnc*, MG1655*rnc* harboring pBetRS1, and either pKAN6B or pRNC1. Total RNA was hybridized with a 5′-^32^P-end-labeled primer (betT+273R) and extended using AMV reverse transcriptase. Sequencing ladders were produced using the same primer used in the PCR DNA, encompassing the *betT* gene as a template. −, no expression; +, endogenous expression; ++, overexpression.
(EPS)

Figure S3 Effects of *betT* deletion on *E. coli* growth upon osmotic stress. The cultures of wild-type and *betT* and/or *proV*-deleted MG1655 were grown in M63 supplemented with 22 mM glucose until an OD_600_ of 0.3 was reached, and either treated with 0.01 M (hypo-osmotic), 0.17 M (normal), or 0.5 M (hyper-osmotic) NaCl with 10 µM choline. Growth was measured by analyzing the cell density (OD_600_) of cultures.
(EPS)

Figure S4 Osmotic regulation of RNase III activity and expression ratio. *E. coli* strains KSC004 and KSC006 were grown in M63 supplemented with 22 mM glucose until an OD_600_ = 0.3 was reached, and then cultures were incubated for a further 3 h in the same medium containing either 0.01 M, 0.17 M, or 0.5 M NaCl with 10 µM choline. Cells were then subjected to β-galactosidase assays (A), and a western blot analysis of RNase III (B). Experiments were performed in triplicate and repeated at least three times.
(TIF)

## References

[1] Amarasinghe AK, Calin-Jageman I, Harmouch A, Sun W, Nicholson AW (2001) *Escherichia coli* ribonuclease III: affinity purification of hexahistidine-tagged enzyme and assays for substrate binding and cleavage. Methods Enzymol 342: 143–158. 11586889 10.1016/s0076-6879(01)42542-0

[2] Court DL, Gan J, Liang YH, Shaw GX, Tropea JE, et al (2013) RNase III: Genetics and Function; Structure and Mechanism. Annu Rev Genet 47: 405–431. 24274754 10.1146/annurev-genet-110711-155618 PMC6311387

[3] Dunn JJ (1976) RNase III cleavage of single-stranded RNA. Effect of ionic strength on the fidelity of cleavage. J Biol Chem 251: 3807–3814. 932008

[4] Zhang H, Kolb FA, Jaskiewicz L, Westhof E, Filipowicz W (2004) Single processing center models for human Dicer and bacterial RNase III. Cell 118: 57–68. 15242644 10.1016/j.cell.2004.06.017

[5] Matsunaga J, Simons EL, Simons RW (1997) *Escherichia coli* RNase III (*rnc*) autoregulation occurs independently of *rnc* gene translation. Mol Microbiol 26: 1125–1135. 9426147 10.1046/j.1365-2958.1997.6652007.x

[6] Gravenbeek ML, Jones GH (2008) The endonuclease activity of RNase III is required for the regulation of antibiotic production by *Streptomyces coelicolor* . Microbiology 154: 3547–3555. 18957607 10.1099/mic.0.2008/022095-0

[7] Sim SH, Yeom JH, Shin C, Song WS, Shin E, et al (2010) *Escherichia coli* ribonuclease III activity is downregulated by osmotic stress: consequences for the degradation of *bdm* mRNA in biofilm formation. Mol Microbiol 75: 413–425. 19943899 10.1111/j.1365-2958.2009.06986.x

[8] Lim B, Sim SH, Sim M, Kim K, Jeon CO, et al (2012) RNase III controls the degradation of *corA* mRNA in *Escherichia coli* . J Bacteriol 194: 2214–2220. 22343302 10.1128/JB.00099-12 PMC3347049

[9] Kavalchuk K, Madhusudan S, Schnetz K (2012) RNase III initiates rapid degradation of *proU* mRNA upon hypo-osmotic stress in *Escherichia coli* . RNA Biol 9: 98–109. 22258144 10.4161/rna.9.1.18228

[10] Lim B, Ahn S, Sim M, Lee K (2014) RNase III controls *mltD* mRNA degradation in *Escherichia coli* . Curr Microbiol 68: 518–523. 24343175 10.1007/s00284-013-0504-5

[11] Song W, Kim YH, Sim SH, Hwang S, Lee JH, et al (2014) Antibiotic stress-induced modulation of the endoribonucleolytic activity of RNase III and RNase G confers resistance to aminoglycoside antibiotics in *Escherichia coli* . Nucleic Acids Res 42: 4669–4681. 24489121 10.1093/nar/gku093 PMC3985665

[12] Andresen PA, Kaasen I, Styrvold OB, Boulnois G, Strøm AR (1988) Molecular cloning, physical mapping and expression of the *bet* genes governing the osmoregulatory choline-glycine betaine pathway of *Escherichia coli.* . J Gen Microbiol 134: 1737–1746. 3065456 10.1099/00221287-134-6-1737

[13] Lamark T, Rokenes TP, McDougall J, Strøm AR (1996) The complex bet promoters of *Escherichia coli*: regulation by oxygen *(*ArcA), choline (BetI), and osmotic stress. J Bacteriol 178: 1655–1662. 8626294 10.1128/jb.178.6.1655-1662.1996 PMC177851

[14] Landfald B (1986) Strøm AR (1986) Choline-glycine betaine pathway confers a high level of osmotic tolerance in *Escherichia coli* . J Bacteriol 165: 849–855. 3512525 10.1128/jb.165.3.849-855.1986 PMC214506

[15] Tondervik A, Strøm AR (2007) Membrane topology and mutational analysis of the osmotically activated BetT choline transporter of *Escherichia coli* . Microbiology 153: 803–813. 17322201 10.1099/mic.0.2006/003608-0

[16] Lamark T, Kaasen I, Eshoo MW, Falkenberg P, McDougall J, et al (1991) DNA sequence and analysis of the bet genes encoding the osmoregulatory choline-glycine betaine pathway of *Escherichia coli* . Mol Microbiol 5: 1049–1064. 1956285 10.1111/j.1365-2958.1991.tb01877.x

[17] Lamark T, Styrvold OB, Strøm AR (1992) Efflux of choline and glycine betaine from osmoregulating cells of *Escherichia coli* . FEMS Microbiol Lett 75: 149–154. 1398030 10.1016/0378-1097(92)90395-5

[18] Styrvold OB, Falkenberg P, Landfald B, Eshoo MW, Bjornsen T, et al (1986) Selection, mapping, and characterization of osmoregulatory mutants of *Escherichia coli* blocked in the choline-glycine betaine pathway. J Bacteriol 165: 856–863. 3512526 10.1128/jb.165.3.856-863.1986 PMC214507

[19] Lee K, Holland-staley CA, Cunningham PR (2001) Genetic approaches to studying protein synthesis: effects of mutations at Psi516 and A535 in *Escherichia coli* 16S rRNA. J nutr 131: 2994S–2004S. 11694635 10.1093/jn/131.11.2994S

[20] Lee K, Varma S, SantaLucia J Jr, Cunningham PR (1997) *In vivo* determination of RNA structure-function relationships: analysis of the 790 loop in ribosomal RNA. J Mol Biol 269: 732–743. 9223637 10.1006/jmbi.1997.1092

[21] Datsenko KA, Wanner BL (2000) One-step inactivation of chromosomal genes in *Escherichia coli* K-12 using PCR products. PNAS 97: 6640–6645. 10829079 10.1073/pnas.120163297 PMC18686

[22] Yeom JH, Go H, Shin E, Kim HL, Han SH, et al (2008) Inhibitory effects of RraA and RraB on RNAse E-related enzymes imply conserved functions in the regulated enzymatic cleavage of RNA. FEMS Microbiol Lett 285: 10–15. 18510556 10.1111/j.1574-6968.2008.01205.x

[23] Yeom JH, Lee K (2006) RraA rescues *Escherichia coli* cells over-producing RNase E from growth arrest by modulating the ribonucleolytic activity. Biochem Biophys Res Commun 345: 1372–1376. 16725107 10.1016/j.bbrc.2006.05.018

[24] Miller JH (1972) Experiments in Molecular Genetics. CSH Laboratory Press, Cold Spring Harbor, NY. 352–355p.

[25] Kim KS, Sim S, Ko JH, Lee Y (2005) Processing of m1 RNA at the 3′ end protects its primary transcript from degradation. J Biol Chem 280: 34667–34674. 16105832 10.1074/jbc.M505005200

[26] Xiao J, Feehery CE, Tzertzinis G, Maina CV (2009) *E. coli* RNase III(E38A) generates discrete-sized products from long dsRNA. RNA 15: 984–991. 19264675 10.1261/rna.1196509 PMC2673077

[27] Wood JM (1999) Osmosensing by bacteria: signals and membrane-based sensors. Microbiol Mol Biol Rev 63: 230–262. 10066837 10.1128/mmbr.63.1.230-262.1999 PMC98963

[28] Wood JM (2006) Osmosensing by bacteria. Sci STKE 357: pe43. 10.1126/stke.3572006pe43 17047223

[29] Cairney J, Booth IR, Higgins CF (1985) Osmoregulation of gene expression in *Salmonella typhimurium*: proU encodes an osmotically induced betaine transport system. J Bacteriol 164: 1224–1232. 3905768 10.1128/jb.164.3.1224-1232.1985 PMC219319

[30] Cairney J, Booth IR, Higgins CF (1985) *Salmonella typhimurium proP* gene encodes a transport system for the osmoprotectant betaine. J Bacteriol 164: 1218–1223. 3905767 10.1128/jb.164.3.1218-1223.1985 PMC219318

[31] Matsunaga J, Dyer M, Simons EL, Simons RW (1996) Expression and regulation of the *rnc* and *pdxJ* operons of *Escherichia coli* . Mol Microbiol 22: 977–989. 8971718 10.1046/j.1365-2958.1996.01529.x

[32] Kim KS, Manasherob R, Cohen SN (2008) YmdB: a stress-responsive ribonuclease-binding regulator of *E. coli* RNase III activity. Genes Dev 22: 3497–3508. 19141481 10.1101/gad.1729508 PMC2607070

[33] Zuker M (2003) Mfold web server for nucleic acid folding and hybridization prediction. Nucleic Acids Res 31: 3406–3415. 12824337 10.1093/nar/gkg595 PMC169194

[34] Mayer JE, Schweiger M (1983) RNase III is positively regulated by T7 protein kinase. J Biol Chem 258: 5340–5343. 6406499

